# Characterization of the family-level *Borreliaceae* pan-genome and development of an episomal typing protocol

**DOI:** 10.1128/mbio.00943-25

**Published:** 2025-05-07

**Authors:** Kalya M. Socarras, Mary C. Marino, Joshua P. Earl, Rachel L. Ehrlich, Nicholas A. Cramer, Joshua C. Mell, Bhaswati Sen, Azad Ahmed, Richard T. Marconi, Garth D. Ehrlich

**Affiliations:** 1 Center for Genomic Sciences, Institute for Molecular Medicine and Infectious Disease, Drexel University College of Medicine 12312, Philadelphia, Pennsylvania, USA; 2 Department of Microbiology and Immunology, Drexel University College of Medicine 12312, Philadelphia, Pennsylvania, USA; 3 Department of Microbiology and Immunology, Virginia Commonwealth University Medical Center 72054, Richmond, Virginia, USA; University of Pittsburgh School of Medicine, Pittsburgh, Pennsylvania, USA

**Keywords:** spirochete, distributed genome hypothesis, pan-genome, supragenome, comparative genomics, phylogenetics, *Borrelia*, *Borreliella*, *Borreliaceae*, taxonomy

## Abstract

The *Borreliaceae* family includes many obligate parasitic bacterial species etiologically associated with a myriad of zoonotic borrelioses, including Lyme disease and vector-borne relapsing fevers. *Borreliaceae* infections are difficult to detect by both direct and indirect methods, often leading to delayed and missed diagnoses. Efforts to improve diagnostics center around the development of molecular diagnostics (MDx), but due to deep tissue sequestration and the lack of persistent bacteremias, even MDx assays suffer from a lack of sensitivity. Additionally, the extensive genomic heterogeneity among isolates, even within the same species, contributes to the lack of assay sensitivity, as single target assays, whether nucleic acid-based or serologically based, cannot provide universal coverage. This within-species heterogeneity is partly due to differences in replicon repertoires and genomic structures that have likely arisen to support the complex *Borreliaceae* life cycle necessary for these parasites to survive in multiple hosts, each with unique immune responses. We constructed a *Borreliaceae* family-level pan-genome and characterized the phylogenetic relationships among the constituent taxa, which supports the recent, although contested, taxonomy of splitting the family into at least two genera. Gene content profiles were created for the majority of the *Borreliaceae* replicons, providing for the first time their unambiguous molecular typing. Our characterization of the *Borreliaceae* pan-genome supports the splitting of the former *Borrelia* genus into two genera and provides for the phylogenetic placement of several non-species designated isolates. Mining this family-level pan-genome will enable the development of precision diagnostics corresponding to gene content-driven clinical outcomes while also providing targets for interventions.

**IMPORTANCE:**

Using whole genome sequencing, we demonstrated that the bacteria that are transmitted by ticks and other arthropod vectors that cause Lyme disease and relapsing fevers, while related, do not belong within the same genus classification. In addition, through characterization of their highly atypical genomic structure, we were able to develop a genetic typing system that will help with future studies of how they cause disease while also providing targets for medical interventions.

## INTRODUCTION

Our development of the distributed genome hypothesis (1–4) led directly to the concept of the bacterial species-level supragenome/pan-genome (5–16). These types of analyses have been extended to the genus level (17–21), and recently to the family level (22, 23). The identification of novel distributed genes and their association with phenotypic characteristics provides for precision diagnostics and targeted characterization of components of the genomic dark matter (19, 24, 25). Despite the progress made in comparative bacterial genomics, there have been relatively few reports examining the agents of spirochetoses, including the *Borreliaceae* (26, 27).

Spirochetoses are characterized as chronic, invasive, debilitating diseases caused by parasitic bacteria within the taxonomic order spirochaetales. Among these diderm helical microbes, the most genomically complex in terms of replicon numbers and heterogeneity are those in the *Borreliaceae* family. This complexity is likely, at least in part, due to the fact that the Borreliaceae have to be well adapted to both vertebrate and arthropod hosts due to their complicated lifecycles. Due to climate change and other anthropomorphic environmental disruptions, the *Borreliaceae* have greatly expanded beyond their traditional geographic epidemiological zones and have developed diverse symbiotic interactions with multiple alternative hosts (28). The true taxonomy of the *Borrelia* genus has been a subject of interest due to the genomic and pathogenic disparities among its member species. A recent taxonomic revision led to the proposed division of the *Borrelia* genus into two distinct genera, *Borreliella* and *Borrelia* (29). In this revised classification, the relapsing fever agents retain the original *Borrelia* taxonomy due to their priority of discovery, and the Lyme borreliosis agents became known as *Borreliella* spirochetes. This work did not include a novel subset of isolates known as the reptile-associated *Borrelia*, which to date have not been well characterized.

It is vital to note that this reclassification has not been without controversy (30–32). In the years since the original proposal, some authors have argued that data other than differences in average nucleotide identity (ANI) do not support the split (30). However, these latter authors were themselves criticized for not fully examining the multi-omic data that provided extensive quantitative data in support of the division (33, 34), thus the controversy continues.

Early genomic studies on *Borreliaceae* spirochetes revealed that they possess a complex genome of a single, linear chromosome terminating in covalently bound hairpin ends, along with multiple (often *n* ≥ 20) single-copy episomes that exist as circular or hairpinned-linear molecules (35, 36).

While the chromosome contains conserved genes linked to essential functions and survival, the individual episomal elements vary widely in size, structure (linear and circular), and gene content, and facilitate host adaptation and/or virulence. Historically, the smaller replicons were found to not be as conserved and were not always retained in long-time cultures. The smaller episomes that were retained or found biologically important were principally categorized by molecular size and structure with only minimal use of gene content.

Despite these limitations, a previous comparative genomic study led to the establishment of a *B. burgdorferi sensu lato* pan-genome (37). This study utilized 23 *Borreliella* genomes, most of which were *B. burgdorferi*, but were of varying assembly quality and plasmid content. This first attempt to create a genus-level pan-genome showed that additional isolates would be necessary to understand the degree of variation among the *Borreliella*. However, until recently, there have been very few pan-genomic publications on these clinically important taxa, and they did not provide information on better targets for these spirochetes classification or mitigation (38, 39). Moreover, there has not been a pan-genome constructed for the *Borrelia* genus nor for the entire *Borreliaceae* family. Thus, there is a need to better understand these pathogens through the construction of a family-level supragenome/pan-genome using the latest error-correcting long-read DNA sequencing methodologies to ensure capture and single-contig assembly of all genomic elements.

## RESULTS

### Study design

All available complete or near-complete high-quality *Borreliaceae* genomes, as of the commencement of this study (*n* = 69) with sequencing coverage greater than 30× and an average N50 (the sequence length of the shortest contig at 50% of the total assembly length) of 900 Kb or above were downloaded from the NCBI prokaryotic genome database and subjected to additional QC analyses for completeness (Table 1). These genomes were then combined with 39 new high-fidelity long-read *Borreliaceae* genomes generated by circular consensus sequencing (CCS) that were chosen to fill in gaps within the taxonomic coverage of the family (Table 2). All pan-genome analyses were conducted from this final curated database of 108 strains (Tables 1 and 2).

**TABLE 1:** NCBI (RefSeq) complete *Borreliaceae* genomes^*a*^

Species	Genus	Group	Isolated from	Vector	Anat	Host	Type	Country	Continent
*Borrelia anserina_BA2*	*Borrelia*	Lb	Direct from host	NA	NA	NA	Chicken	NA	NA
*Borrelia anserina_Es*	*Borrelia*	Rf	Direct from host	NA	NA	*Gallus gallus domesticus*	Chicken	United States	North America
*Borrelia coriaceae_Co53*	*Borrelia*	Rf	Direct from host	*Ornithodoros coriaceus*	Soft	*Ornithodoros coriaceus*	Tick	NA	NA
*Borrelia hermsii_CC1*	*Borrelia*	Rf	Direct from tick	*Ornithodoros hermsii*	soft	*Ornithodoros hermsii*	Chipmunk	United States	North America
*Borrelia hermsii_DAH*	*Borrelia*	Rf	Direct from tick	*Ornithodoros hermsii*	soft	*Ornithodoros hermsii*	Tick	United States	North America
*Borrelia hermsii_DAH_2E7*	*Borrelia*	Rf	Direct from host	NA	NA	*Homo sapiens*	Human	United States	North America
*Borrelia hermsii_HS1*	*Borrelia*	Rf	Direct from tick	*Ornithodoros hermsii*	Soft	*Ornithodoros hermsii*	Tick	United States	North America
*Borrelia miyamotoi_CA17_2241*	*Borrelia*	Rf	Direct from tick	*Ixodes pacificus*	Hard	*Ixodes pacificus*	Tick	United States	North America
*Borrelia miyamotoi_CT13_2396*	*Borrelia*	Rf	Direct from tick	*Ixodes scapularis*	Hard	*Ixodes pacificus*	Tick	United States	North America
*Borrelia miyamotoi_CT14D4*	*Borrelia*	Rf	Tick_via_host	*Ixodes scapularis*	Hard	*Homo sapiens*	Human	United States	North America
*Borrelia miyamotoi_CZ_F190E*	*Borrelia*	Rf	Direct from tick	*Ixodes ricinius*	Hard	*Ixodes ricinius*	Tick	Czech Republic	Europe
*Borrelia miyamotoi_CZ_F1E*	*Borrelia*	Rf	Direct from tick	*Ixodes ricinius*	Hard	*Ixodes ricinius*	Tick	Czech Republic	Europe
*Borrelia miyamotoi_Hk004*	*Borrelia*	Rf	Direct from tick	*Ixodes persculatus*	Hard	*Ixodes persculatus*	Tick	Japan	Asia
*Borrelia miyamotoi_HT24*	*Borrelia*	Rf	Direct from tick	*Ixodes persculatus*	Hard	*Ixodes persculatus*	Tick	Japan	Asia
*Borrelia miyamotoi_HT31*	*Borrelia*	Rf	Direct from tick	*Ixodes persculatus*	Hard	*Ixodes persculatus*	Tick	Japan	Asia
*Borrelia miyamotoi_Izh_4*	*Borrelia*	Rf	Direct from host	NA	NA	*Homo sapiens*	Human	Russia	Eurasia
*Borrelia miyamotoi_Izh_5*	*Borrelia*	Rf	Direct from host	NA	NA	*Homo sapiens*	Human	Russia	Eurasia
*Borrelia miyamotoi_LB_2001*	*Borrelia*	Rf	Direct from tick	*Ixodes scapularis*	Hard	*Mus musculus*	Mouse	United States	North America
*Borrelia miyamotoi_M12C4*	*Borrelia*	Rf	Direct from tick	*Ixodes persculatus*	Hard	*Ixodes persculatus*	Tick	Mongolia	Asia
*Borrelia miyamotoi_M15A8*	*Borrelia*	Rf	Direct from tick	*Ixodes persculatus*	Hard	*Ixodes persculatus*	Tick	Mongolia	Asia
*Borrelia miyamotoi_M20E6*	*Borrelia*	Rf	Direct from tick	*Ixodes persculatus*	Hard	*Ixodes persculatus*	Tick	Mongolia	Asia
*Borrelia miyamotoi_MYK1*	*Borrelia*	Rf	Direct from tick	*Ixodes persculatus*	Hard	*Ixodes persculatus*	Tick	Japan	Asia
*Borrelia miyamotoi_MYK2*	*Borrelia*	Rf	Direct from tick	*Ixodes persculatus*	Hard	*Ixodes persculatus*	Tick	Japan	Asia
*Borrelia miyamotoi_MYK3*	*Borrelia*	Rf	Direct from tick	*Ixodes persculatus*	Hard	*Ixodes persculatus*	Tick	Japan	Asia
*Borrelia miyamotoi_MYK4*	*Borrelia*	Rf	Direct from tick	*Ixodes persculatus*	Hard	*Ixodes persculatus*	Tick	Japan	Asia
*Borrelia miyamotoi_MYK5*	*Borrelia*	Rf	Direct from tick	*Ixodes persculatus*	Hard	*Ixodes persculatus*	Tick	Japan	Asia
*Borrelia miyamotoi_NB103/1*	*Borrelia*	Rf	Direct from tick	*Ixodes persculatus*	Hard	*Ixodes persculatus*	Tick	Japan	Asia
*Borrelia miyamotoi_Y14T1*	*Borrelia*	Rf	Direct from tick	*Ixodes persculatus*	Hard	*Ixodes persculatus*	Tick	Japan	Asia
*Borrelia miyamotoi_Y14T18*	*Borrelia*	Rf	Direct from tick	*Ixodes ovatus*	Hard	*Ixodes ovatus*	Tick	Japan	Asia
*Borrelia miyamotoi_Y15T1*	*Borrelia*	Rf	Direct from tick	*Ixodes persculatus*	Hard	*Ixodes persculatus*	Tick	Japan	Asia
*Borrelia miyamotoi_Yekat_1*	*Borrelia*	Rf	Direct from host	NA	NA	*Homo sapiens*	Human	Russia	Eurasia
*Borrelia miyamotoi_Yekat_6*	*Borrelia*	Rf	Direct from host	NA	NA	*Homo sapiens*	Human	Russia	Eurasia
*Borrelia parkeri_HR1*	*Borrelia*	Rf	Direct from tick	*Ornithodoros parkeri*	Soft	*Otospermophilus beecheyi*	Squirrel	United States	North America
*Borrelia recurrentis_A1*	*Borrelia*	Rf	Direct from host	NA	NA	*Homo sapiens*	Human	Ethiopia	Eurasia
*Borrelia sp_A_FGy1*	*Borrelia*	Rf	Direct from tick	*Amblyomma longiroste*	Hard	*Amblyomma longiroste*	NA	NA	NA
*Borrelia sp_tHM16w*	*Borrelia*	Unk	Direct from tick	*Haemaphysalis megaspinosa*	Hard	*Haemaphysalis megaspinosa*	NA	Japan	Asia
*Borrelia turcica_IST7*	*Borrelia*	MR	Tick_via_host	*Hyalomma aegyptium*	Hard	NA	Tortoise	Turkey	Europe/Asia
*Borrelia turicatae_bt_91E135*	*Borrelia*	Rf	Tick_via_host	*Ornithodoros_turicata*	Soft	*Mus musculus*	Mouse	United States	North America
*Borrelia turicatae_BTE5EL*	*Borrelia*	Rf	Direct from host	NA	NA	*Homo sapiens*	Human	United States	North America
*Borreliella afzelii_BO23*	*Borreliella*	Lb	Direct from host	NA	NA	*Homo sapiens*	Human	Germany	Europe
*Borreliella afzelii_HLJ01*	*Borreliella*	Lb	Direct from host	NA	NA	*Homo sapiens*	Human	NA	NA
*Borreliella afzelii_K78*	*Borreliella*	Lb	Direct from tick	NA	NA	*Homo sapiens*	Human	Austria	Europe
*Borreliella afzelii_PKo_GCF_000165595_2*	*Borreliella*	Lb	Direct from host	NA	NA	*Homo sapiens*	Human	Germany	Europe
*Borreliella afzelii_PKo_GCF_000222835_1*	*Borreliella*	Lb	Direct from host	NA	NA	*Homo sapiens*	Human	Germany	Europe
*Borreliella afzelii_S13*	*Borreliella*	Lb	Direct from tick	*Ixodes nipponensis*	Hard	*Ixodes nipponensis*	Tick	NA	NA
*Borreliella afzelii_Tom3107*	*Borreliella*	Lb	Direct from tick	*Ixodes persculatus*	Hard	*Ixodes persulcatus*	Tick	Russia	Eurasia
*Borreliella bavariensis_NT24*	*Borreliella*	Lb	Direct from tick	*Ixodes persculatus*	Hard	*Ixodes persculatus*	Tick	Japan	Asia
*Borreliella bavariensis_PBi*	*Borreliella*	Lb	Direct from host	NA	NA	*Homo sapiens*	Human	Germany	Europe
*Borreliella bissettii_DN127*	*Borreliella*	Lb	Direct from tick	*Ixodes pacificus*	Hard	*Ixodes pacificus*	Tick	United States	North America
*Borreliella burgdorferi_3028*	*Borreliella*	Lb	Direct from host	NA	NA	*Homo sapiens*	Human	United States	North America
*Borreliella burgdorferi_B17_2013*	*Borreliella*	Lb	Direct from tick	*Ixodes ricinus*	Hard	*Ixodes ricinus*	Tick	Belarus	Europe
*Borreliella burgdorferi_B31*	*Borreliella*	Lb	Direct from tick	*Ixodes scapularis*	Hard	*Ixodes scapularis*	Tick	United States	North America
*Borreliella burgdorferi_B31_NRZ*	*Borreliella*	Lb	Direct from tick	*Ixodes scapularis*	Hard	*Ixodes scapularis*	Tick	United States	North America
*Borreliella burgdorferi_B331*	*Borreliella*	Lb	Direct from host	NA	NA	*Homo sapiens*	Human	United States	North America
*Borreliella burgdorferi_B500*	*Borreliella*	Lb	Direct from host	NA	NA	*Homo sapiens*	Human	United States	North America
*Borreliella burgdorferi_JD1*	*Borreliella*	Lb	Direct from tick	*Ixodes scapularis*	Hard	*Ixodes scapularis*	Tick	United States	North America
*Borreliella burgdorferi_MM1*	*Borreliella*	Lb	Direct from host	NA	NA	*Peromyscus leucopus*	Mouse	United States	North America
*Borreliella burgdorferi_N40*	*Borreliella*	Lb	Direct from tick	*Ixodes scapularis*	Hard	*Ixodes scapularis*	Tick	United States	North America
*Borreliella burgdorferi_PAbe*	*Borreliella*	Lb	Direct from host	NA	NA	*Homo sapiens*	Human	Germany	Europe
*Borreliella burgdorferi_PAli*	*Borreliella*	Lb	Direct from host	NA	NA	*Homo sapiens*	Human	Germany	Europe
*Borreliella burgdorferi_ZS7*	*Borreliella*	Lb	Direct from tick	*Ixodes scapularis*	Hard	*Ixodes scapularis*	Tick	United States	North America
*Borreliella garinii_20047*	*Borreliella*	Lb	Direct from tick	*Ixodes ricinius*	Hard	*Ixodes ricinius*	Tick	France	Europe
*Borreliella garinii_BgVir*	*Borreliella*	Lb	Direct from tick	*Ixodes persculatus*	Hard	*Ixodes persculatus*	Tick	NA	NA
*Borreliella garinii_isolate: 20047, CIP 103362*	*Borreliella*	Lb	Tick_via_host	*Ixodes ricinus*	Hard	*Homo sapiens*	Human	France	Europe
*Borreliella mayonii_MN14-1420*	*Borreliella*	Lb	Direct from host	NA	NA	*Homo sapiens*	Human	United States	North America
*Borreliella mayonii_MN14-1539*	*Borreliella*	Lb	Direct from host	NA	NA	*Homo sapiens*	Human	United States	North America
*Borreliella valaisiana_Tom4006*	*Borreliella*	Lb	Direct from tick	*Ixodes persculatus*	Hard	*Ixodes persculatus*	Tick	Russia	Eurasia
*Borreliella valaisiana_VS116*	*Borreliella*	Lb	Direct from tick	*Ixodes ricinius*	Hard	*Ixodes ricinus*	Tick	Switzerland	Europe
*Borreliella maritima_CA690*	*Borreliella*	Lb	Direct from tick	*Ixodes sninipalpis*	Hard	*Ixodes sninipalpis*	Tick	United States	North America
*Candidatus Borrelia tachyglossi_Bc_F10_1268*	*Borrelia*	MR	Tick_via_host	*Bothrioroton concolor*	Hard	NA	Echidna	Australia	Australia

^*a*^ Anat* = tick species anatomy type (hard or soft); Rf = relapsing fever; Lb = Lyme borreliosis; MR = nonotreme and reptile associated; NA = information is not available. Note: although *Ixodes scapularis*, often referred to as the deer tick, is the most frequent arthropod vector of Lyme disease in the endemic U.S. Northeast, its principal hosts and the principal reservoirs of *Borreliella burgdorferi* are small rodents, including the white-footed mouse, *Peromyscus leucopus*, and hence this is why this study and others chiefly include isolates from these small mammals.

**TABLE 2:** Newly sequenced *Borreliaceae* spirochetes^*a*^

Species	Genus	Group	Isolated from	Vector	Anat	Host	Type	Country	Continent
*Borrelia hermsii_HS1d*	*Borrelia*	Rf	Direct from tick	*Ornithodoros hermsii*	Soft	*Ornithodoros hermsii*	Tick	United States	North America
*Borrelia turicatae_6232*	*Borrelia*	Rf	Direct from tick	*Ornithodoros parkeri*	Soft	*Ornithodoros parkeri*	Tick	NA	NA
*Borrelia turicatae_RUM*	*Borrelia*	Rf	Direct from host	NA	NA	*Homo sapiens*	Human	United States	North America
*Borreliella afzelii_B_5_92*	*Borreliella*	Lb	Direct from host	NA	NA	*Homo sapiens*	Human	Norway	Europe
*Borreliella afzelii_ECM1*	*Borreliella*	Lb	Direct from host	NA	NA	*Homo sapiens*	Human	Sweden	Europe
*Borreliella afzelii_IP21*	*Borreliella*	Lb	Direct from tick	*Ixodes persculatus*	Hard	*Ixodes persculatus*	Tick	Russia	Eurasia
*Borreliella afzelii_RIP3*	*Borreliella*	Lb	Direct from tick	*Ixodes persculatus*	Hard	*Ixodes persulcatus*	Tick	NA	NA
*Borreliella afzelii_VS461*	*Borreliella*	Lb	Unknown	NA	NA	NA	NA	Switzerland	Europe
*Borreliella burgdorferi_B408*	*Borreliella*	Lb	Direct from host	NA	NA	*Homo sapiens*	Human	United States	North America
*Borreliella burgdorferi_B479*	*Borreliella*	Lb	Direct from host	NA	NA	*Homo sapiens*	Human	United States	North America
*Borreliella burgdorferi_BO23d*	*Borreliella*	Lb	Direct from host	NA	NA	*Homo sapiens*	Human	Germany	Europe
*Borreliella burgdorferi_c77632*	*Borreliella*	Lb	Tick_via_host	NA	NA	NA	Mouse	NA	NA
*Borreliella burgdorferi_c77636*	*Borreliella*	Lb	Tick_via_host	NA	NA	NA	Mouse	NA	NA
*Borreliella burgdorferi_c77643*	*Borreliella*	Lb	Tick_via_host	NA	NA	NA	Mouse	NA	NA
*Borreliella burgdorferi_c77644*	*Borreliella*	Lb	Tick_via_host	NA	NA	NA	Mouse	NA	NA
*Borreliella burgdorferi_CA12*	*Borreliella*	Lb	Direct from tick	*Ixodes pacificus*	Hard	*Ixodes pacificus*	Tick	United States	North America
*Borreliella burgdorferi_CA2*	*Borreliella*	Lb	Direct from tick	*Ixodes neotomae*	Hard	*Ixodes neotomae*	Tick	United States	North America
*Borreliella burgdorferi_HB19*	*Borreliella*	Lb	Direct from host	NA	NA	*Homo sapiens*	Human	United States	North America
*Borreliella burgdorferi_LDP60*	*Borreliella*	Lb	Direct from host	NA	NA	*Homo sapiens*	Human	United States	North America
*Borreliella burgdorferi_LDP73*	*Borreliella*	Lb	Direct from host	NA	NA	*Homo sapiens*	Human	United States	North America
*Borreliella burgdorferi_LDP74*	*Borreliella*	Lb	Direct from host	NA	NA	*Homo sapiens*	Human	United States	North America
*Borreliella burgdorferi_MI_2*	*Borreliella*	Lb	Direct from host	NA	NA	*peromyscus gossypinus*	Mouse	United States	North America
*Borreliella burgdorferi_N40d*	*Borreliella*	Lb	Direct from tick	*Ixodes scapularis*	hard	*Ixodes scapularis*	Tick	United States	North America
*Borreliella burgdorferi_NCH_1*	*Borreliella*	Lb	Direct from host	NA	NA	*Homo sapiens*	Human	United States	North America
*Borreliella burgdorferi_NY_13*	*Borreliella*	Lb	Direct from tick	*Ixodes ricinus*	hard	*Ixodes ricinus*	Tick	Norway	Europe
*Borreliella burgdorferi_Phoe*	*Borreliella*	Lb	Direct from host	NA	NA	*Homo sapiens*	Human	Germany	Europe
*Borreliella burgdorferi_veery*	*Borreliella*	Lb	Direct from host	NA	NA	*catharus fuscescens*	Bird	United States	North America
*Borreliella burgdorferi_VS134*	*Borreliella*	Lb	Direct from tick	*Ixodes ricinus*	Hard	*Ixodes ricinus*	Tick	Switzerland	Europe
*Borreliella garinii_20047d*	*Borreliella*	Lb	Direct from tick	*Ixodes ricinus*	Hard	*Ixodes ricinus*	Tick	France	Europe
*Borreliella garinii_AO1*	*Borreliella*	Lb	Direct from host	NA	NA	*Homo sapiens*	Human	NA	NA
*Borreliella garinii_DK26*	*Borreliella*	Lb	Direct from host	NA	NA	*Homo sapiens*	Human	Germany	Europe
*Borreliella garinii_FRG*	*Borreliella*	Lb	Direct from tick	*Ixodes ricinus*	Hard	*Ixodes ricinus*	Tick	NA	NA
*Borreliella garinii_G2*	*Borreliella*	Lb	Direct from host	NA	NA	*Homo sapiens*	Human	Germany	Europe
*Borreliella garinii_Jem5*	*Borreliella*	Lb	Direct from host	NA	NA	*Homo sapiens*	Human	Japan	Asia
*Borreliella garinii_N34*	*Borreliella*	Lb	Direct from tick	*Ixodes ricinius*	Hard	*Ixodes ricinus*	Tick	Germany	Europe
*Borreliella garinii_PbaeII*	*Borreliella*	Lb	Direct from host	NA	NA	*Homo sapiens*	Human	Germany	Europe
*Borreliella garinii_Pfin*	*Borreliella*	Lb	Direct from host	NA	NA	*Homo sapiens*	Human	Germany	Europe
*Borreliella garinii_VSBP*	*Borreliella*	Lb	Direct from tick	*Ixodes ricinius*	Hard	*Ixodes ricinius*	Tick	Switzerland	Europe
*Borreliella_MOK_3a*	*Borreliella*	Lb	Direct from tick	*Ixodes dentatus*	Hard	*Ixodes dentatus*	Tick	United States	North America

^*a*^ Anat* = tick species anatomy type (hard or soft); Rf = relapsing fever; Lb = Lyme borreliosis; MR = Monotreme and reptile associated; NA = information is not available.

### Pacific biosciences sequel I whole-genome sequencing and validation

A modified gel-plug DNA isolation and pulse field gel electrophoresis (PFGE) analysis was performed on the *Borreliaceae* type strains (*Borreliella burgdorferi* strain B31 and *Borrelia hermsii* strain HS1) to precisely determine the number of replicons and to serve as a control for the extraction and sequencing of the multiple replicons that make up the *Borreliaceae* genomes (40). Using the same DNAs, we performed whole genome sequencing on a Pacific Biosciences (PacBio) Sequel I employing single molecule error-correcting circular consensus sequencing. Quality assurance measures of the WGS included assembling the linear chromosomes into a single contig ≥0.9 Mb and identifying each of the other assembled contigs (9 to 200 Kb) as corresponding to one of the PFGE replicons.

**Fig 1:**
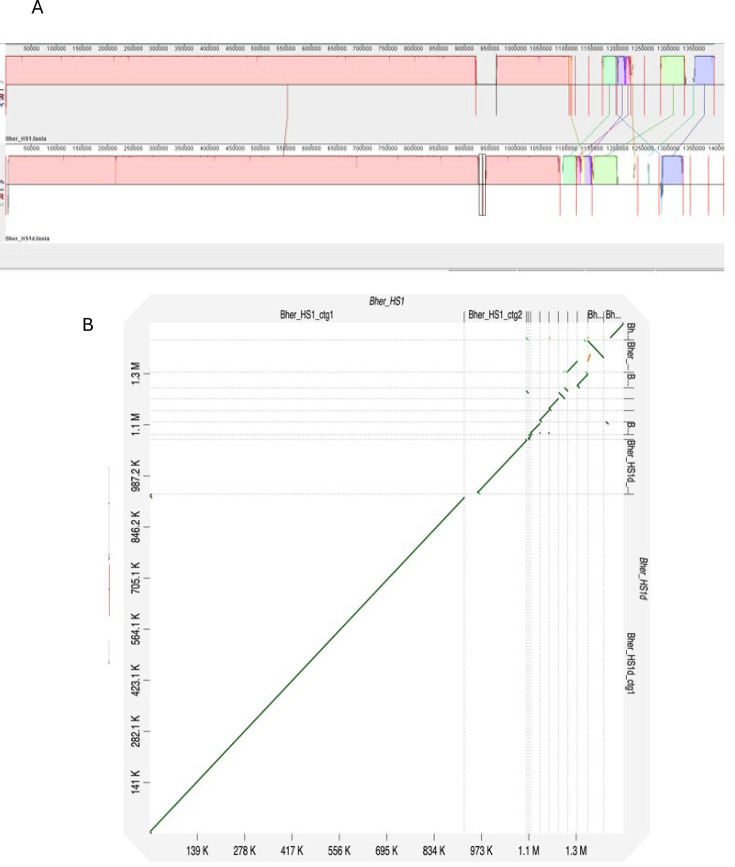
*In silico* comparison of the genomes of two *B. hermsii* HS1 strains shows homology throughout several replicons and indicates regions of heterogeneity. (**A**) Alignments of a pair of *B. hermsii* HS1 strains show nearly identical genomes. Similar pairwise locally collinear blocks are indicated in the same colors (pink to pink, blue to blue, etc.). Contig breaks are indicated by red lines. (**B**) D-Genies dot plot of whole-genome complete reference *B. hermsii* strain HS1 (query, *x*-axis) to our sequenced *B. hermsii* strain HS1d (target, *y*-axis). Overall percent similarity was indicated from red (low = 0) to green (high = 100%). All contigs of the two whole genome sequences are portrayed in the figure. Each contig pair is separated by a dotted line. The position of the matches between strains is indicated by colored dots and lines (yellow, orange, and green). Green lines perpendicular to the diagonal are the result of the linear episomal hairpin ends.

We first performed comparative genomic analyses of the published sequences of the *Borreliella burgdorferi* B31 and *Borrelia hermsii* HS1 strains with our lab-sequenced genomes of the same two strains, designated as B31d and HS1d, respectively. This was done to determine if all replicons of both these *Borreliaceae* species representing the two genera were present and fully sequenced via our protocols (41, 42). We confirmed that our whole genome sequencing was comparable to previous work by aligning a reference and our sequence of *B. hermsii* strain HS1 (Fig. 1A). Using a progressive Mauve alignment of the reference strain and our sequenced *B. hermsii* strain HS1d, multiple locally collinear blocks (LCBs) were noted across the entire length of the genome. The largest LCB showed high homology between our sequence and the reference’s linear chromosome and its large linear plasmid. The linear chromosomes contained 100 single nucleotide polymorphisms (SNPs), and the large linear plasmids had five SNPs. The smaller contigs from both genomes were either partially aligned or had no clear matches, likely in part due to progressive Mauve being unable to track duplications and the shared gene content between some plasmids (43). The estimated total number of SNPs between the two HS1 genomes was ~1,000. These differences likely stem from small duplications near the telomeres of the linear plasmids since progressive Mauve is ill-suited to handle them. Additionally, novel plasmids within the newly sequenced *B. hermsii* strain HS1d may have arisen from recombination events to which *Borreliaceae* plasmids are prone (38, 44), or alternatively due to plasmid loss during the culturing of the original HS1 isolate. A second alignment using D-Genies corroborated these findings (Fig. 1B).

Finally, whole genome alignments of the reference *B. hermsii* strain HS1 and our sequenced *B. hermsii* strain HS1d confirmed their overall similarity but also demonstrated that they are not identical, particularly with respect to the episomal elements (data not shown). Some differences in the episomal elements may be accounted for by genes encoding proteins undergoing antigenic variation or by the variable cassettes which drive genetic conversion.

### Coverage of covalently bound hairpin ends of *Borreliaceae* episomes

**Fig 2:**
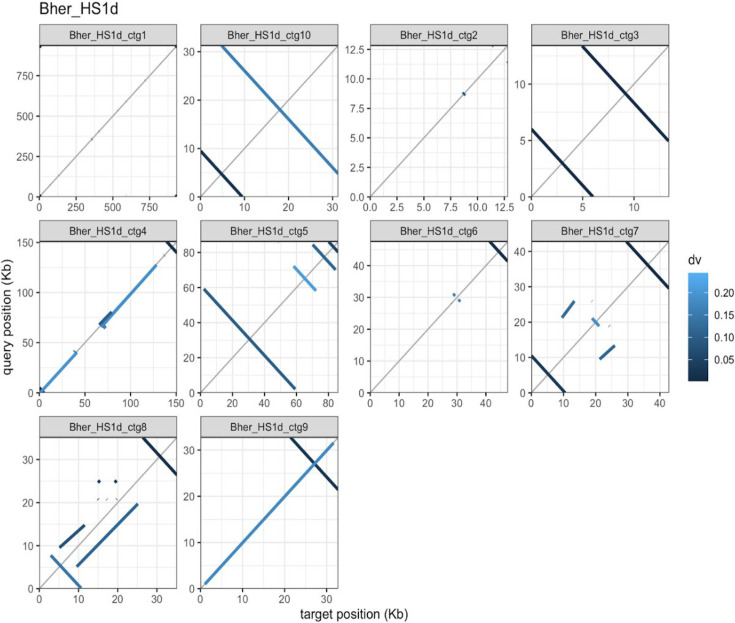
Self-alignment of *B. hermsii* strain HS1d contigs reveals inverted repeats at linear contig ends. A faceted pafr dot plot of each contig within the *B. hermsii* strain HS1d whole-genome sequence. The overall percent similarity was indicated from light blue (low = 0) to dark blue (high = 100%). All contigs were represented within each individual facet of the plot. Within each plot, *x*- and *y*-axes are the overall lengths of the compared contigs.

In our initial alignments, several of the reference *B. hermsii* strain HS1 plasmids exhibited homology to the middle of our newly sequenced HS1d contigs (Fig. 1A). These homologies were identified as short flanking inverted repeats at the ends of many HS1d contigs that were not included in any LCB with the HS1 reference genome (Fig. 1 and 2). This was determined to be an artifact of the hairpin ends of the linear replicons and the circular sequencing approach we used. These inverted repeat sequences arise from sequencing the hairpin present within the ends of all linear *Borreliaceae* replicons (35, 38, 45). This occurs because the natural hairpin mimics the SMRTbell primer ligated onto the sheared chromosomal DNA fragments used for CCS sequencing on the PacBio platform. Thus, these artifacts arise from a terminal linear chromosomal fragment that has a SMRTbell primer at one end (the sheared end) and the *Borreliaceae* hairpin at the other end.

**Fig 3:**
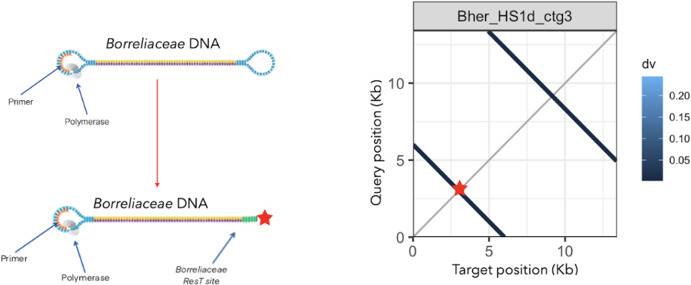
Blast search of *B. hermsii* strain HS1 linear contigs reveals ResT sites located at the ends of replicons. A graphic depiction of Pacific Biosciences long-read sequencing platform and modified prepared *Borreliaceae* template (replicon image modified from Pacific Biosciences 2022 using Procreate). Alongside the sequencing schematic is one of the faceted plots of *B. hermsii* strain HS1d (contig 3). This plot shows the percent similarity of the replicon along its entire length with regions of similarity removed. Perpendicular lines are inverted repeats. The overall percent similarity was indicated from light blue (low = 0) to dark blue (high = 100%). All contigs were represented within each individual facet of the plot.

To confirm this hypothesis, we paired the contigs and aligned them using the D-Genies program (Fig. 1B). This analysis identified regions with the inverted repeats present at the very ends of some HS1d contigs that indeed were the termini of linear replicons. Further inspection of the *B. hermsii* strain HS1d contigs using minimap2 and subsequent plotting with pafr confirmed this artifact occurred at both ends of the linear plasmids sequenced using the Pacbio platform (Fig. 2) (45–47). Further proof of this phenomenon was obtained by inspecting the heterogeneous ends of these contigs for the presence of the telomere resolvase (ResT) Box 3 TATA motif (35, 38, 45, 48, 49) found in all *Borreliaceae* species (Fig. 3). These motifs were identified on the linear *B. hermsii* strain HS1d contigs as well as within all of our sequenced *Borreliaceae* cohort of genomes. To ensure that these inverted repeat sequences would not skew downstream comparative genomic analyses, each contig that contained a ResT Box 3 motif was trimmed down.

### Average nucleotide identity (ANI) analyses support a digeneric *Borreliaceae*

**Fig 4:**
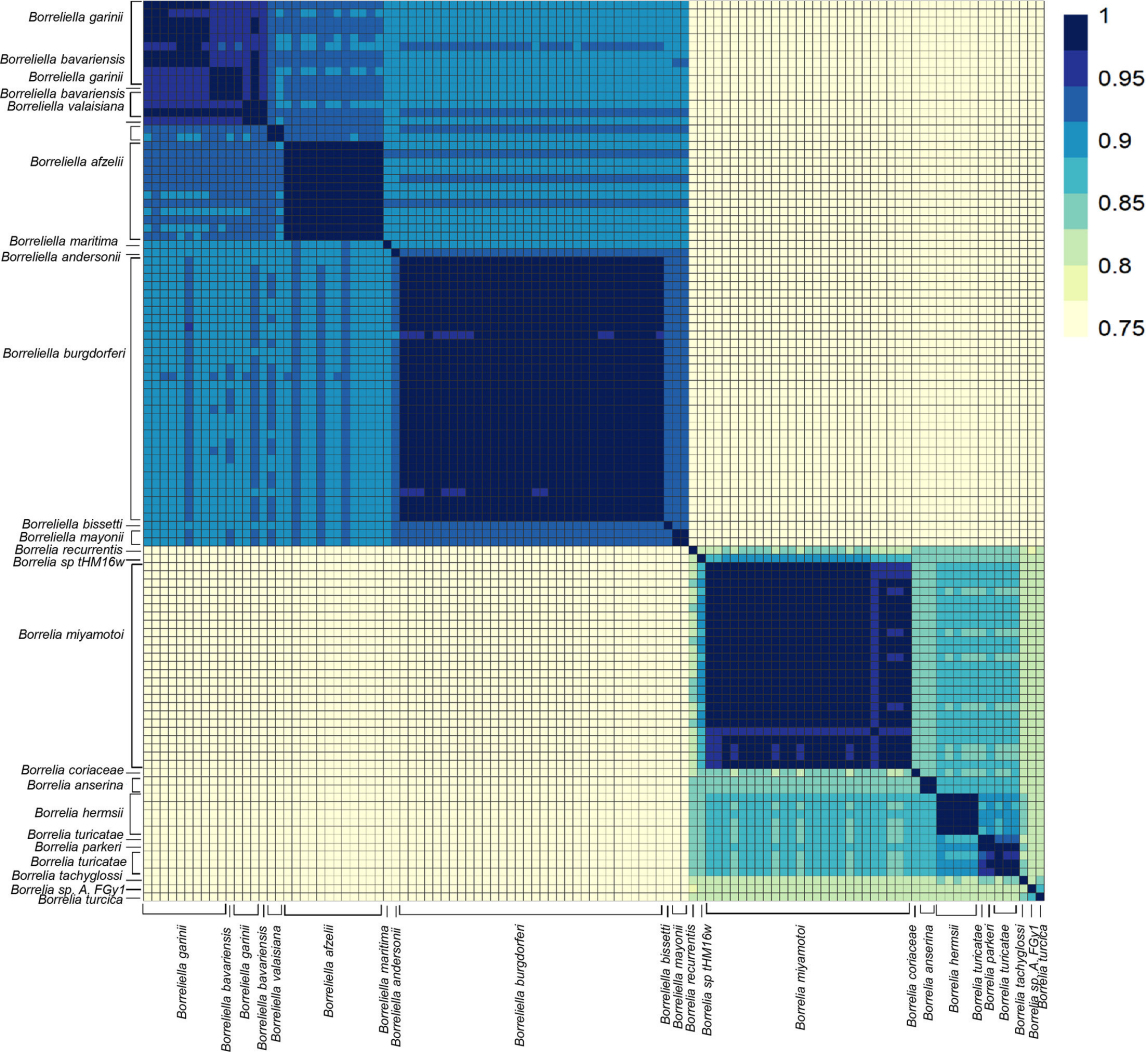
Average nucleotide identity (ANI) of all *Borreliaceae* species and strains examined demonstrated genera-specific clustering. The range of ANI is from least (yellow) to greatest (blue). Each species is represented on both the *x*- and *y*-axes. The diagonal line represents identity. The dendrograms were made with complete-linkage hierarchical clustering. The key color is noted on the right-hand side. The annotations indicate the generic groupings.

The ANI among all *Borreliaceae* genome assemblies was computed using the pyani average nucleotide identity blast method (BLASTN+). These analyses revealed that the species members of the original single *Borrelia* genus are clearly separated into two groups corresponding to the proposed (50) *Borrelia* and *Borreliella* genera (Fig. 4). Thus, our findings concur with the division of the former *Borrelia* genus into two genera, and henceforth we will refer to the Lyme borreliosis-causing spirochetes as *Borreliella* and the relapsing fever-causing spirochetes as *Borrelia*. Additionally, we included in the analysis two non-species designated *Borrelia* spirochetes, B_sp_HM_thM16W and B_sp_FGy1, which are thought to be part of the reptile-associated group (51–55) that were demonstrated to be *sui generis* but clustered more closely with the *Borreliella* genus than the *Borrelia*.

**Fig 5:**
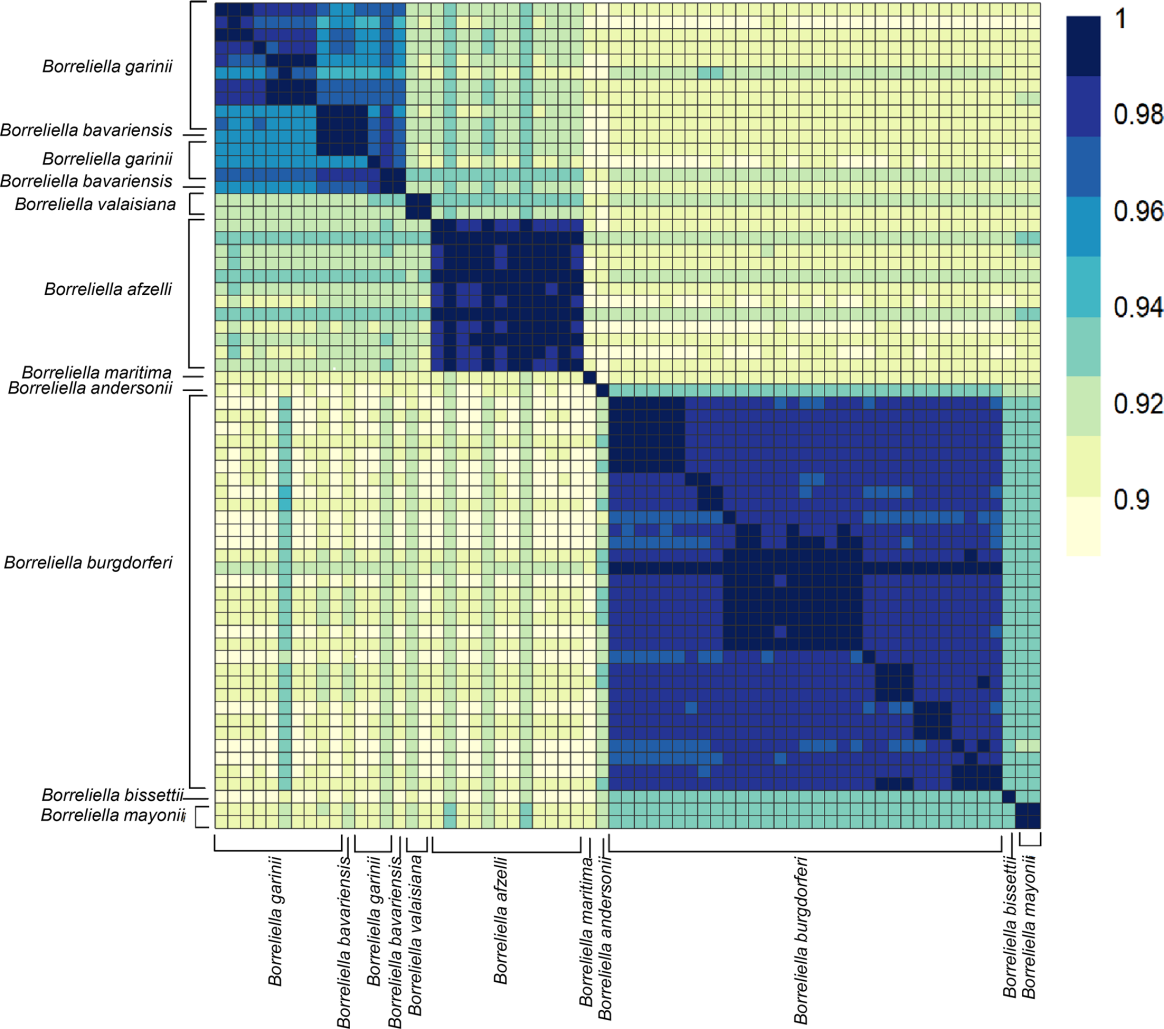
Average nucleotide identity (ANI) analyses of *Borreliella* genomes show a high degree of similarity amongst the major species of the genus. The range of ANI is from least (yellow) to greatest (blue). Each of the *Borreliella* spirochetes is represented on both the *x*- and *y*-axes. The diagonal line represents identities. The dendrograms were made with complete-linkage hierarchical clustering. The color bar key is noted on the right-hand side. The annotations indicate the species group.

Further inspection of the 65 *Borreliella* strains included in the analyses, covering nine named species, demonstrated that the vast majority have been placed within the correct taxa, with essentially all pairwise strain analyses within a species showing >96% ANI (Fig. 5). All the *B. burgdorferi* and *B. afzelii* were correctly assigned. One of the two strains typed as *B. bavariensis* clustered within the *B. garinii*. The other typed with one of the two strains typed as *B. valaisiana*, forming a small *sui generis* group which may correspond to a hybrid species between *B. garinii* and *B. afzelii* as the hierarchical clustering placed them between the proposed parent species. These findings are in line with previous reports that had suggested that *B. bavariensis* was a subspecies of *B. garinii*. *B. maritima* was equidistant between *B. afzelii* and *B. burgdorferi*. In addition, we characterized the phylogenetic relationship of several novel isolates included in this study. These included the lagomorph isolate, *Borreliella andersonii*, which at the time of its discovery was misclassified as a *B. burgdorferi* strain (56) but was later reclassified as *B. andersonii* (57). In our ANI analyses, the *B. andersonii* strain MOK_3a clustered closely to *B. burgdorferi and B. maritima*. Of the remaining three *Borreliella* species (*B. andersonii*, *B. bissetii*, and *B. mayonii*), all were most closely related to *B. burgdorferi,* but each was sufficiently distant to warrant their species designations.

**Fig 6:**
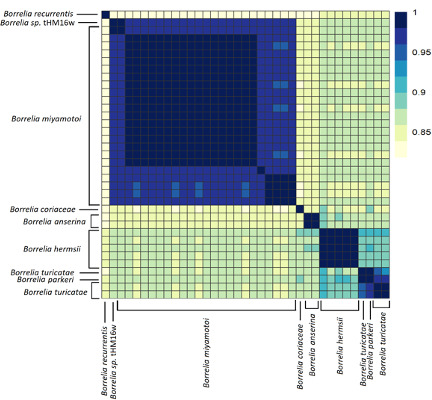
Average nucleotide identity (ANI) of *Borrelia* genomes shows separation of the major species. The range of ANI is from least (yellow) to greatest (blue). Each *Borrelia* spirochete was represented on both the *x*- and *y-*axes. The diagonal line represents identities. The dendrograms were made with complete-linkage hierarchical clustering. The color bar key is noted on the right-hand side. The annotations indicate the species group.

**Fig 7:**
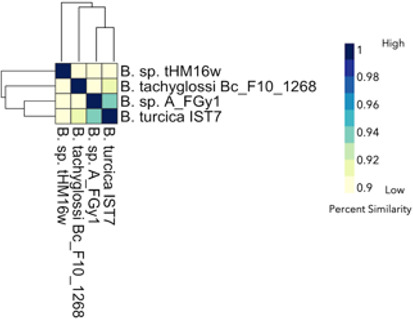
Average nucleotide identity (ANI) of *Borreliaceae* genomes from monotreme/reptile-associated isolates (which included isolates from ticks recovered from reptiles) suggests their placement within the family tree. The range of ANI is from least (yellow) to greatest (blue). Each isolate is represented on both the *x*- and *y*-axes. The diagonal line represents identities. The dendrograms were made with complete-linkage hierarchical clustering. The color bar key is noted on the right-hand side. The annotations indicate the species group

Compared with the *Borreliella*, the *Borrelia* spirochetes displayed greater genetic heterogeneity. The *B. miyamotoi* strains clustered strongly together, displaying an average pairwise ANI of 97% (Fig. 6) but were distinct from the cluster of species containing *B. hermsii*, *B. parkeri*, and *B. turicatae*. The reptile-associated *Borrelia* species were both remarkably distant from these other major relapsing fever spirochetes and highly divergent among themselves, with only B_sp_FGy1 and *B. turcica* strains sharing any substantial similarity at 94% (Fig. 7). Through ANIb (blast), we determined that these two genomes bracket the *Borrelia* genera (Fig. 4). The isolate B_sp_HM_thM16w was also divergent from the group and was closest to *B. recurrentis* (Fig. 4 and 6).

**Fig 8:**
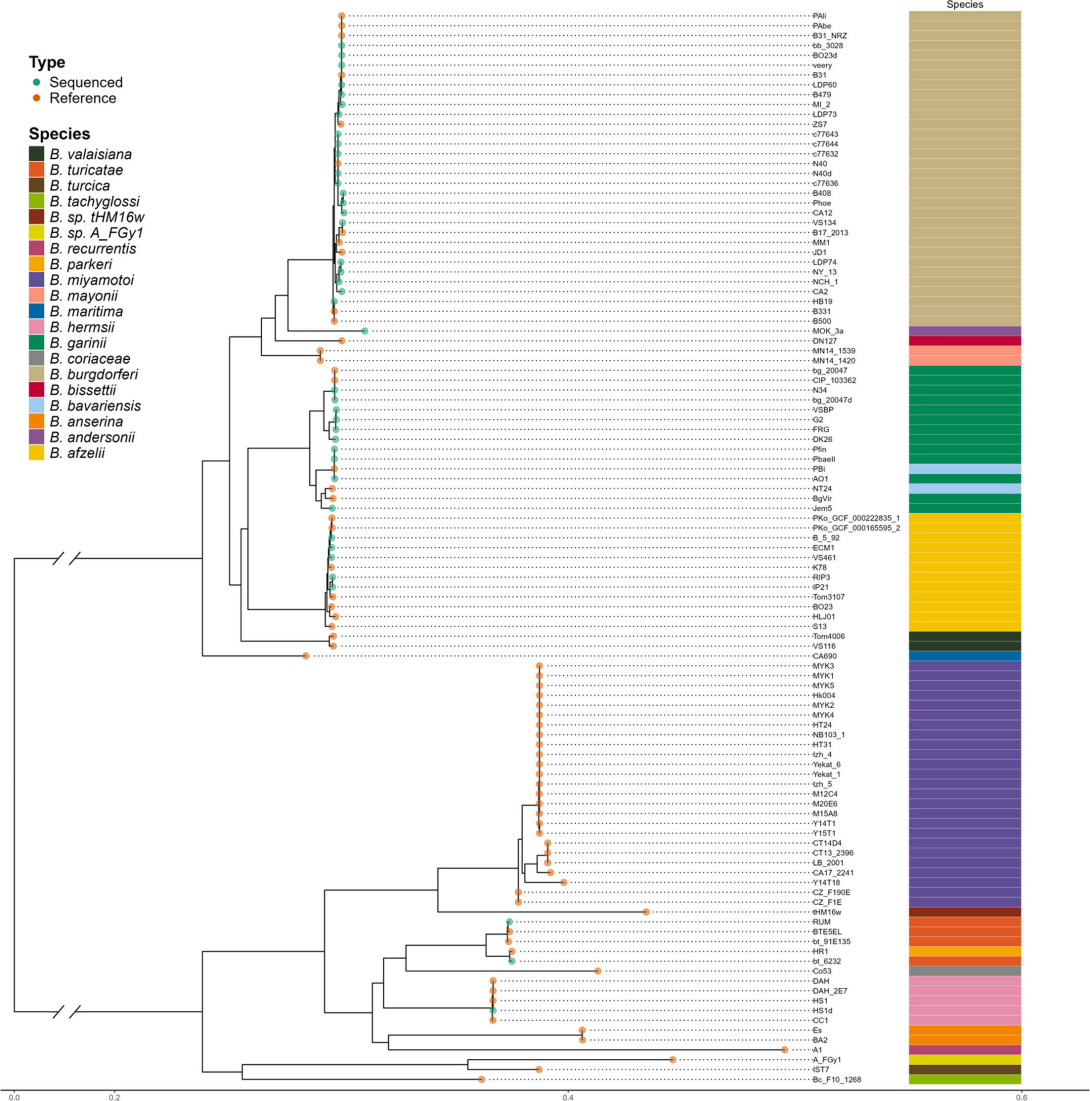
Midpoint-rooted single-copy core gene tree of the *Borreliaceae* shows the division between the *Borreliella* and *Borrelia* genera. The origin of the spirochete is indicated by the color of the nodes: blue (Virginia Commonwealth University); red (NCBI). The annotation bar indicates the species.

Following our initial ANI analysis, the evolutionary relationships among all Borreliaceae strains included in the study were characterized by the construction of maximum likelihood phylogeny (Fig. 8). This revealed both a division among the *Borreliaceae* spirochetes and the same clustering for *Borreliella* and *Borrelia* spirochetes. Interestingly, we observed that some more recently identified *Borreliella and Borrelia* isolates cluster within their respective genera. This is most clearly illustrated with *B. andersonii*, which, based on the single-copy core phylogeny, was most closely related to *B. burgdorferi* and *B. bissettii*. Other novel genomes, like those seen within the reptile-associated *Borrelia* spirochetes, stood apart from the majority of the *Borrelia* genus as had been previously seen in the ANI. Only the novel *B. sp strain thHMw* intercalated within *B. miyamotoi* and *B. hermsii* strains. The rest of the reptile-associated spirochetes formed a separate clade within *Borrelia* spirochetes.

### *Borreliaceae* family-level pan-genome

**Fig 9:**
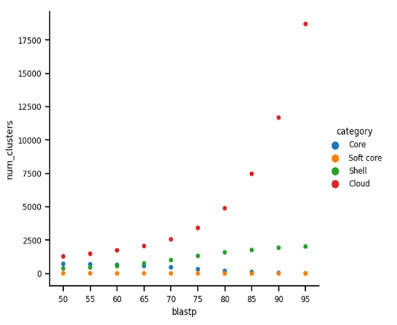
The gene cluster frequency of the *Borreliaceae* family pan-genome reveals a large pan-genome. Each colored circle signifies different components of the pan-genome [core (blue), soft-core (orange), shell (green), and cloud (red)]. The *x*-axis signifies the BlastP percent homology threshold. The *y*-axis signifies the number of genes within the pan-genome.

Following our initial ANI analyses, we wanted to understand how similar the genera were based on their overall gene similarities. The percentage of shared genes within the family was approximately 50% (Fig. 9). To begin to understand which genes are shared between the different genera, the *Borreliaceae* family-level pan-genome was multiply calculated at 5% intervals of increasing similarity (Fig. 9). At 75% BlastP, clear separation between the core and cloud components of the *Borreliaceae* pan-genome is seen (Fig. 9). At the 75% BlastP threshold, there are 322 core genes, eight soft-core genes, 1,352 genes in the shell, and 3,424 genes in the cloud. There are 1,253 unique genes for all 109 isolates included in this study. Interestingly, the *Borreliaceae* pan-genome had a different composition when analyzed with the EggNOG algorithm. Through this second approach, EggNOG estimates that *Borreliaceae* contains 997 unique genes for 109 isolates. Furthermore, its pan-genome distribution consists of 577 core, 30 soft-core, 204 shell, and 213 cloud genes. Although each of these pan-genome analysis tools was given the same data set, they yielded different results. These discrepancies are due to underlying differences in the alignment methods used by the algorithms. EggNOG relies upon alignment to a database of proteins which have been pre-clustered, rather than relying on a specific blast threshold. Additionally, EggNOG largely ignores genes of unknown function rather than labeling them as “unknown,” which artificially decreases the number of unique genes.

Previously, a *Borreliella* genospecies complex pan-genome was constructed through the use of 22 genomes comprised of *B. burgdorferi, B. afzelii,* and *B. garinii* (37). In this work, they found that the *Borreliella* pan-genome was open. To ascertain if this was true within our own curated collection of *Borreliaceae* genomes, we next tested for changes in the pan-genome using both data acquired from Roary at 75% BlastP and EggNOG (Fig. S1). It is evident that the number of gene clusters present within each genus-specific pan-genome compartment is subject to change at the incorporation of a novel pan-genome. This suggests that the family-level pan-genome is open with a growing distributed component reminiscent of what was found within the early *Borreliella* comparative genomic study.

**Fig 10:**
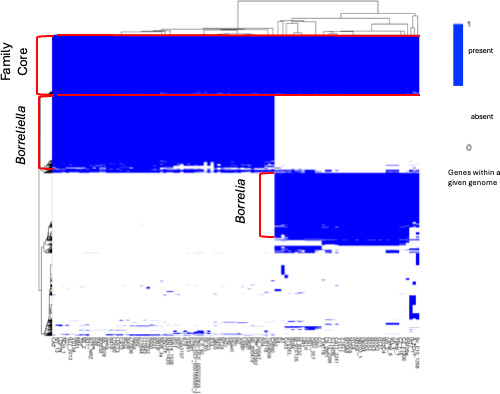
*Borreliaceae* gene presence/absence heatmap. Genes are colored blue if present and white if absent. Homologs were defined as having Roary BlastP of ≥75%. The *y*-axis represents gene clusters within the *Borreliaceae* pan-genome. The *x*-axis depicts all *Borreliaceae* isolates included in the study. The red annotations indicate the gene groups. The *Borreliaceae* pan-genome has a relatively small core, with each of the two genera-defined core genomes being more than twice as large as the family core genome. Only the most prevalent of the distributed genes (not present in either of the three core genomes) are included in the heatmap.

Next, we visualized the *Borreliaceae* pan-genome using a heat map for all included isolates (Fig. 10). The family-level core genome consists of 488 genes possessed by all isolates in both genera. Interestingly, the family-level distributed pan-genome is fragmented into multiple components. This division reveals two additional groups of genes that, on further inspection, were identified as the core genes of either the *Borreliella* or *Borrelia* genera.

### The *Borreliaceae* episomes can be grouped through gene content

**Fig 11:**
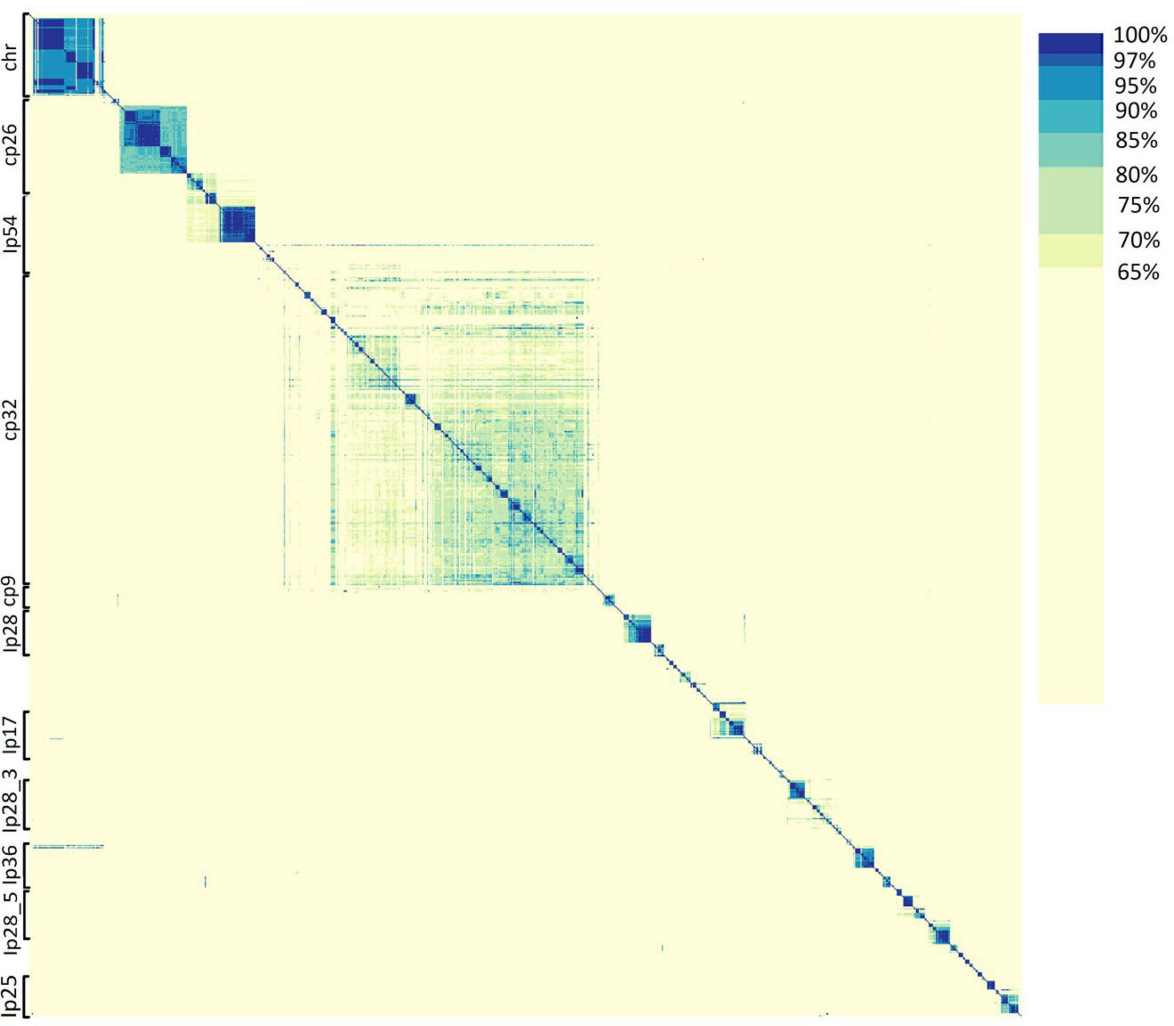
Large-scale average nucleotide identity (ANI) of *Borreliaceae* replicons reveals definitive groupings across the family. The range of ANI is from least (yellow) to greatest (blue). Each of the Borreliaceae replicons is represented on the *x*- and *y*-axes based on complete-linkage hierarchical clustering. The annotations indicate the replicon group.

*Borreliaceae* spirochetes thrive throughout their complex lifecycles during which they undergo multiple transmissions between phylogenetically distinct hosts including arthropod vectors and multiple classes of vertebrates. Their ability to survive in various hosts is dependent upon a large set of distributed genes that are mostly encoded on episomal replicons. These episomes have traditionally been typed primarily based on molecular size, structure (linear or circular), and alleles of proteins in the PFam32 family (58). To ensure proper episomal identification, this system needs to be expanded to include information on the entire gene repertoire of all available episomes. To this end, we ran a pyani ANIb on approximately 1,800 *Borreliaceae* replicons from our curated genome collection. As pyani ANIb may not factor all gene content within the replicons, we also employed a Hadamard matrix (which interprets identity and coverage simultaneously) for this purpose. In the Hadamard matrix heatmap, multiple subgroups for nearly all of the *Borreliaceae* family replicons were identified (Fig. 11).

The most similar episomes clustered closely together. The three most conserved groups are the *Borreliella* chromosome, cp26, and lp54. Other *Borreliella* episomal groups with relatively higher percentages of similarity were lp25 and lp36. Interestingly, there was very broad and diffuse clustering for the clinically significant family of cp32 plasmids which are known to contain genes encoding host-specific proteins, indicating both their relatedness and the very high degrees of heterogeneity necessary to ensure survival during a lifecycle that includes multiple hosts. Unlike cp32, the lp28 episomes do not likely represent a single family of replicons as they did not all cluster together and instead separated out into smaller clusters.

For the *Borrelia* genus, a high degree of similarity was seen among the chromosomes as well as for the large linear plasmids. Additionally, species-specific groupings amongst the episomes were also observed. This is best illustrated in *B. miyamotoi* strains lp6 and lp12. It is important to note that while the reptile-associated *Borrelia* spirochetes were included in these analyses, they were the only isolates to have extraordinarily dissimilar replicons. This is likely attributable to the incompleteness of the B_sp_A_FGy1 genome as indicated by its large number of constituent contigs.

## DISCUSSION

The debilitating diseases caused by *Borreliaceae* spirochetes have remained on the periphery of societal memory for centuries. Despite our current knowledge of the etiological agents of these spirochetoses, there is still a paucity of information on how each disease is caused and why their presentations are so variable among individuals. In the work presented in this study, multiple pressing points within the field of *Borreliaceae* comparative genomics were addressed to help provide a framework going forward to be able to associate microbial genotypes with disease phenotypes in the host.

The first topic we addressed was the composition of the *Borreliaceae* family. It was recently proposed that the previous single genus, *Borrelia*, be divided into two genera based on differences in overall genomic content (29, 34). The well-studied and more widely known Lyme borreliosis spirochetes were given the genus name of *Borreliella*, while the older and less clinically studied species complex comprising the relapsing fever spirochetes retained the *Borrelia* designation. Since the establishment of this proposed dichotomy, there has been animated discussion among scientists both for and against the split (30–34).

Pairwise ANI analyses of all 108 sequenced isolates (*N* = 11,556 genome comparisons) in this study revealed a sharp division between the two proposed genera, indicating that the recent taxonomic re-classification is correct. Even isolates from within each of the genera often did not share sequence similarities above 85%. While it may be true that the ANI cut-off can vary based on species as seen with *Stenotrophomonas maltophila, Escherichia* spp., or *Lactobacillus* spp., our results do not support including the Lyme borreliosis spirochetes and relapsing fever spirochetes within a single genus (59, 60).

Additional analyses revealed that the recently sequenced novel spirochaetal species B_sp_FGy1 and B_sp_HM_thM16w clustered most closely with *B. anserina* and *B. recurrensis,* respectively. These two genomes clustered within the known reptile-associated *Borreliaceae*. As these isolates are novel, it is unknown if they are pathogenic to humans.

We constructed a family-level pan-genome to characterize the similarities and differences between the two genera concerning gene content. In doing so, we found that the family-level *Borreliaceae* core genome was very small, consisting of only 488 genes, but that each of the genus-level core genomes were each more than double that size and that the family-level pan-genome is still open. This indicates that there are large numbers of distributed genes still to be discovered. The number of core genes possessed by both genera were similar.

It was previously known that there is some similarity between *Borreliella* and *Borrelia* replicons with the *Borrelia* replicons containing regions of similarity to the *Borreliella* cp26 and lp54 (27, 44, 61, 62). To better place the various replicons within a gene-functional framework, we established a system for typing the *Borreliaceae* plasmids. Previously, these episomes have been typed based on size, structure, and the presence of specific alleles of particular protein families found on many of the plasmids (26, 58). While this technique helped somewhat in the identification of the many *Borreliaceae* episomes, it did little to identify them by the biological functions they encode. In our work, all of the available episomes for each of the isolates in our data set were used in an ANI analysis via pyani. Doing so for over 1,800 replicons resulted in a complex matrix that did little to clarify how these episomes related to each other (Fig. 11). However, in building a Hadamard matrix from these analyses, it was possible to visualize each of the known plasmids with respect to their overall hierarchical clustering, even for the most heterogenous replicon, cp32. Thus, we have established a universal *Borreliaceae* plasmid typing system based on gene content that captures all of the replicons produced via our PacBio-based long-read sequencing protocols.

Finally, we identified an artifact of sequencing linear bacterial replicons with hairpin termini with the PacBio SMRTbell technology. In our sequencing, it was noted that some of the many linear episomes had regions of heterogeneity. After closer inspection, it became apparent that these regions were inverted repeats that could emerge as artifacts in long-read sequencing platforms (38, 63). These regions were identified as the telomeric ends of all linear *Borreliaceae* replicons, a feature not unlike those seen in higher eukaryotes (41, 45, 64, 65). To prevent false inflation in the genes within our data set, we searched for and confirmed the location of the ResT Box 3 site (49, 65). From this, we have begun to build the first system to handle these *Borreliaceae* long-read sequencing artifacts that could be applied to any replicon with similar features. These data also suggest that raw PacBio circular consensus sequences may contain hidden information about hairpin and cruciform DNA structures in other genomes.

This work on the *Borreliaceae* family-wide pan-genome is pivotal and the first of its kind. More sequencing is required to ensure that the pan-genome is truly reflective of all *Borreliaceae* spirochetes. Furthermore, more work is required for the typing of all *Borreliaceae* episomes. This family-level pan-genome analysis provides new tools and information in a field that requires more insight on how to handle such evasive and persistent pathogens. More importantly, this comparative genomic work can serve as the basis for identifying and characterizing novel taxon-specific conserved targets for the development of precision diagnostics and therapeutics.

### Conclusions

Through the application of multiple comparative genomic methods including phylogenetics, ANI, gene content, and core genome analyses, we demonstrate that the *Borreliaceae* are composed of at least two genera. We also developed a gene-based plasmid typing protocol to replace the current confusing method that relies solely on size and topography. This approach to episomal typing revealed an extremely high degree of allelic heterogeneity in the plasmid(s) that contain the genes that encode the major host-interacting proteins, which are reflective of the extremely broad host range of these parasitic bacteria. Additional analyses with a larger number of genomes will be required to determine if the reptile-associated strains and other underrepresented groups in the current analysis define additional genera.

## MATERIALS AND METHODS

### *Borreliaceae* pan-genome project design

This study included all available reference *Borreliaceae* genomes obtained from NCBI RefSeq on 17 October 2021. The downloaded genomes were checked for completeness, sequencing platform, sequencing coverage, and if the average number of contigs greatly superseded the number of included replicons. For example, selected genomes were disqualified if more than two or three contigs were needed for a single replicon. Additionally, they were run through a QC pipeline to ensure usability in later studies. The remainder of the genomes was used henceforth. Novel isolates collected by collaborators at Virginia Commonwealth University were then sequenced at the Center for Genomic Sciences core facility at Drexel University College of Medicine. These newly sequenced members of the *Borreliaceae* are included in BioProject PRJNA1026537.

### *Borreliaceae* culturing

All *Borreliella* and *Borrelia* isolates were cultivated in 50 mL of BSK-II media supplemented with 6% or 12% rabbit serum (Sigma, MA), respectively. The culture was then monitored using wet mounts and dark-field microscopy. Once spirochetes grew to the concentration of 5 × 10^7^ cells/mL at late log phase, they were harvested by centrifugation, resuspended in 1× sterile PBS, and then recovered by centrifugation.

### Pulse-field Gel Plasmid Verification

DNA isolation of selected *Borreliaceae* isolates was done through a modified BioNano plug prep (Bionano Genomics, Bionano Prep Cell Culture DNA Isolation Protocol) (Bionano Genomics, CA). Cultured *Borreliaceae* were pelleted at 5,000×*g* for 10 min and sent overnight to the Center for Genomic Sciences at Drexel University College of Medicine. Received pellets were immediately thawed, resuspended with a total volume of 66 µL of cell buffer, and then 40 µL of 2% agarose was added. The contents were mixed 10 times via pipette, and then 100 µL of each sample was aliquoted into a plug mold. The filled plug mold was incubated at 4°C for 15 min before starting protein digestion. This process was done by placing the plug into a solution of 167 µL proteinase K (Qiagen, DE) and 2.5 mL Lysis buffer, then incubated for 2 h at 50°C in a thermomixer at intermittent mixing. After incubation, the digestion solution was replaced and incubated overnight on the thermomixer as described above.

Following the overnight incubation, plugs were allowed to cool at room temp for 5 min and then we added 50 µL of RNase A before placing the plugs in the thermomixer at 37°C with intermittent mixing. Plugs were then washed 3 times with 10 mL 1× Wash buffer before being washed once more with 10 mL and set on an orbital shaker for 15 mins at 180 RPM. This latter step was repeated three more times. DNA recovery was initiated by adding 10 mL of 1× TE buffer to wash plugs and shaking at 180 RPM for 15 min a total of 5 times. Cleaned plugs were then inserted into a 1% low melt agarose gel and run on a pulse-field gel at 5V for 21 h. Pulse field gel was stained with ethidium bromide and visualized under UV.

### *In silico Borreliaceae* replicon verification

The *Borreliaceae* genome assembly pairs were aligned through progressiveMauve (v.2.4.0), NUCmer (MUMer 3.0) and D-Genies (v1.4.0) (43, 66, 67). Additionally, each spirochete genome FASTA file that had been used for the above initial validation work was split by contig, imported into R, and then aligned to itself using pafr (v 0.0.2) (46).

### *Borreliaceae* DNA extraction

Once received, frozen *Borreliaceae* pellets were thawed on ice and centrifuged at 13,000 rpm for 1 min. Following pelleting, the remaining solvent was discarded before the pellets were resuspended in 200 µL of 1X PBS, pipette mixed, and transferred to a 2 mL bead beating tube (Matrix E) (MP Biomedicals, CA). Following the transfer, 20 µL of proteinase K (Qiagen, DE) was added, and cells were homogenized using a SPEX 1600 MiniG (Fisher Scientific, MA) for 1 min at 1500 Hz. Subsequent DNA extraction was performed using the Qiagen DNeasy Blood & Tissue Kit according to the manufacturer’s instructions (Qiagen, DE).

### DNA preparation and sequencing

Extracted *Borreliaceae* DNA was quantified following ThermoFisher Scientific 1X dsDNA HS kit per the manufacturer’s instructions (Biotium Inc, CA) on Qubit. DNA from each Borrelial/Borrelia specimen was prepped with the SMRTbell Template Prep Kit 2.0 (Pacific Biosciences, CA) to make PacBio SMRTbell libraries with barcodes sourced from the Barcoded Overhang Adaptor Kit 8A and 8B (Pacific Biosciences, CA). The sequencing primers were then annealed and bound to Polymerase 3.0 using the Sequel Binding Kit 3.0 (Pacific Biosciences, CA). The final bound complex was then purified and later sequenced on PacBio Sequel I using the SMRT Cell M1 v3 tray (Pacific Biosciences, CA). The spike-in controls for each PacBio Sequel I run were from the Internal Control Kit 3.0 (Pacific Biosciences, CA).

### Genome assembly, annotation, and pan-genome construction

*Borreliaceae* isolates that were sequenced on the PacBio Sequel I platform were processed using pbcromwell (v 1.0.4) (Pacific Biosciences, CA). All the barcoded data were demultiplexed using pb_demux_subreads and assembled using pb_assembly_microbial. The base modification motifs for each assembly were computed using pb_basemods. The species of each genome was verified using Taxator and GTDB-Tk (v1.7.0) with refpack (v r202). All reference and sequenced *Borreliaceae* assemblies were annotated with Prokka (v 1.11), and then homologous genes were clustered with Roary (v 3.5.1) (68, 69). The BlastP threshold was calculated consecutively at 5% intervals to determine the threshold. The final BlastP threshold, 75%, was selected based on the separation of the core pan-genome from the cloud and accessory pan-genomes. All CDS identified by Prokka were also annotated using EggNOG (70).

### Phylogenetic tree construction

Single-copy core genes of each *Borreliaceae* genome were aligned using MAFFT (v7.490-1) and then the concatenated alignment was run through Randomized Accelerated Maximum Likelihood (RaxML) to generate a Newick string (71, 72). The resulting file was then ported into RStudio (v2022.07.2+576), a tree was generated using ape and then visualized using ggtree (v3.15) R packages (73, 74).

### Average nucleotide identity and percent similarity or difference analysis

All pairs of *Borreliaceae* genomes, both sequenced and downloaded, were checked for overall similarity by ANI using the pyani (v2.12) with the ANIb method (75). The resulting whole-genome ANI matrix (or Hadamard matrix) was read into RStudio (v.4.1.1), clustered with adapted RaxML phylogeny, and visualized with pheatmap (v1.0.12).

The *Borreliaceae* percent gene similarity was calculated using RStudio in a pairwise fashion for each isolate in this study. The final list of shared percent identity was then pivoted wider to generate a matrix. Any pairs that did not have a gene percent similarity value were assigned a value of 0. The final matrix was then clustered with hclust and visualized with pheatmap (v1.0.12).

### *Borreliaceae* pan-genome mapping

To find pan-genome distribution, all Roary at BlastP 75% or EggNOG gene clusters were imported into R (70). Then, each gene was associated with a corresponding strain and/or replicon before being used to construct a presence-absence matrix. This matrix was then visualized in pheatmap to see the gene presence and absence across all species used in this study. To determine if the *Borreliaceae* pan-genome was either open or closed, the composition was checked by shuffling strains, incorporating them one at a time into the analysis, and then calculating the core/distributed component. The combinations of strains incorporated were shifted prior to being plotted in each iteration.

## Data Availability

The sequencing data used in this study are available under Bioproject PRJNA1026537.

## SUPPLEMENTAL MATERIAL

Figure S1 mbio.00943-25-s0001.tiff Roary vs. EggNOG pan-genomes.

Legend mbio.00943-25-s0002.docx Supplemental figure legend.

ASM does not own the copyrights to Supplemental Material that may be linked to, or accessed through, an article. The authors have granted ASM a non-exclusive, world-wide license to publish the Supplemental Material files. Please contact the corresponding author directly for reuse.
