# Supplementary figures and images for: Characterization of the family-level Borreliaceae pan-genome and development of an episomal typing protocol
Source: mBio. 2025 May 7;16(6):e00943-25. doi: 10.1128/mbio.00943-25 (PMC12153284; doi:10.1128/mbio.00943-25)

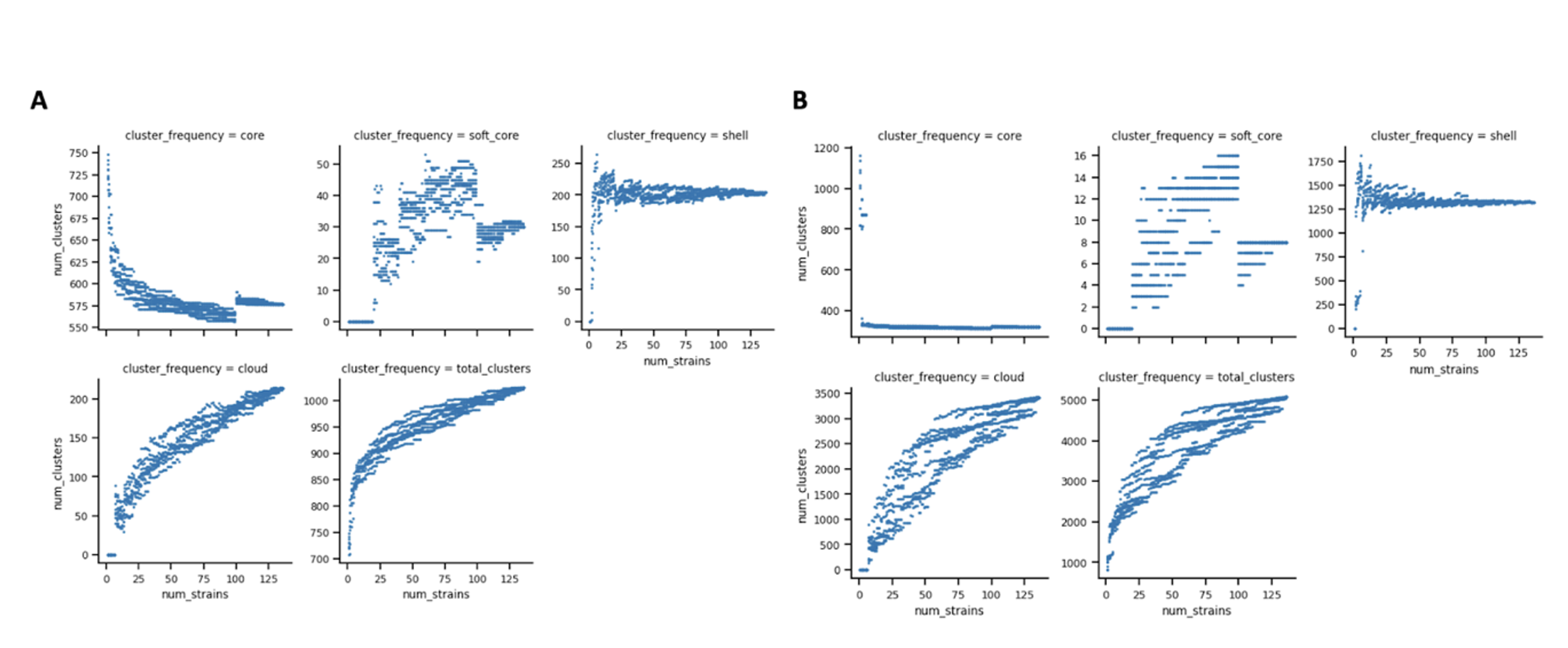

Supplement: Figure S1 — Roary vs. EggNOG pan-genomes. [file mbio.00943-25-s0001.tiff]
