# Supplementary material for: Characterization of the family-level Borreliaceae pan-genome and development of an episomal typing protocol
Source: mBio. 2025 May 7;16(6):e00943-25. doi: 10.1128/mbio.00943-25 (PMC12153284; doi:10.1128/mbio.00943-25)
Supplement: Legend — Supplemental figure legend. [file mbio.00943-25-s0002.docx]

**Supplementary Figure S1.** Roary and EggNOG depict an expanding Borreliaceae pangenome over the number of included isolate genomes. X- axis represents the number of isolates and Y-axis represents the number of gene clusters within pangenome. Each panel-faceted plot is dedicated to different components of the pangenome: A) Roary BlastP 75% was chosen as the threshold. Each light blue dot indicates an incorporated isolate. B) EggNOG depiction of pangenome fluctuation with each incorporated genome.
